# Physical Activity Producing Low, but Not Medium or Higher, Vertical Impacts Is Inversely Related to BMI in Older Adults: Findings From a Multicohort Study

**DOI:** 10.1093/gerona/glx176

**Published:** 2017-09-19

**Authors:** Ahmed Elhakeem, Kimberly Hannam, Kevin C Deere, April Hartley, Emma M Clark, Charlotte Moss, Mark H Edwards, Elaine Dennison, Tim Gaysin, Diana Kuh, Andrew Wong, Cyrus Cooper, Rachel Cooper, Jon H Tobias

**Affiliations:** 1 Musculoskeletal Research Unit, School of Clinical Sciences, University of Bristol, UK; 2 MRC Lifecourse Epidemiology Unit, University of Southampton, UK; 3 MRC Unit for Lifelong Health and Ageing, University College London, UK

**Keywords:** Accelerometer, Body mass index, Fat mass, Lean mass, Body composition

## Abstract

**Background:**

High impact physical activity (PA) is thought to improve skeletal health, but its relation to other health outcomes are unclear. We investigated associations between PA impact magnitude and body mass index (BMI) in older adults.

**Methods:**

Data were taken from the Cohort for Skeletal Health in Bristol and Avon (COSHIBA), Hertfordshire Cohort Study, and MRC National Survey of Health and Development. Vertical acceleration peaks from 7-day hip-worn accelerometer recordings were used to classify PA as low (0.5 < *g* < 1.0*g*), medium (1 < *g* < 1.5*g*), or higher (≥1.5*g*) impact. Cohort-specific associations of low, medium, and higher impact PA with BMI were examined using linear regressions and estimates combined using random-effects meta-analysis.

**Results:**

A total of 1182 participants (mean age = 72.7 years, 68% female) were included. Low, medium, and higher impact PA were inversely related to BMI in initial models. After adjustment for confounders and other impacts, low, but not medium or higher, impacts were inversely related to BMI (−0.31, *p* < .001: overall combined standard deviation change in BMI per doubling in the number of low impacts). In adjusted analyses of body composition measured by dual-energy X-ray absorptiometry in COSHIBA, low, but not medium or higher, impacts were inversely related to total body fat mass (−0.19, *p* < .001) and android:gynoid fat mass ratio (−0.16, *p* = .01), whereas high impact PA was weakly and positively associated with lean mass (0.05, *p* = .06).

**Conclusions:**

Greater exposure to PA producing low magnitude vertical impacts was associated with lower BMI and fat mass at older age. Low impact PA may help reduce obesity risk in older adults.

Habitual levels of physical activity (PA) are inversely related to the risk of obesity; epidemiological studies reveal similar relationships irrespective of whether PA is ascertained by questionnaire or more objective assessment using accelerometry (1–3). PA is generally quantified in terms of energy consumption, reflecting a combination of PA duration and intensity, the latter being categorized according to Metabolic Equivalent of Tasks as light, moderate, and vigorous based on counts per minute (cpm) thresholds applied to outputs from Actigraph accelerometers (4). As expected, for any given duration of PA, more intense PA as defined in this way is associated with a greater reduction in fat mass compared with the same duration of less intense PA (1).

PA is beneficial for several other health systems including the skeleton; however, in contrast to obesity, PA effects on bone are mediated by deformations caused by impacts (5). Therefore, to evaluate relationships between PA and skeletal outcomes, the former needs to be characterized according to impact level as opposed to energy consumption, including quantification of exposure to rare but highly osteogenic high impacts (6). We developed a method for characterizing exposure to vertical impacts according to impact level, based on raw Y-axis recording from accelerometers (7) and validated this in older individuals attending an exercise class (8). On applying this method in a population-based cohort of older women, we found that the positive relationship between habitual PA and lower limb bone strength in older women is solely explained by exposure to vertical impacts ≥1.5 g, despite their rarity (9).

Direct signals have been postulated between bone and regulation of energy balance (10). Therefore, impacts might also influence the risk of obesity independently of energy consumption and lead to greater benefit than that predicted based on energy consumption alone. On the other hand, impacts from ground reaction forces are influenced by intrinsic structure of muscles, tendons, and ligaments, which may have little to do with energy consumption. Hence, whereas our recent findings suggest that PA producing higher impacts should be encouraged to optimize skeletal health in older individuals, to what extent this approach will benefit other health outcomes such as obesity is currently unclear.

In the present study, we investigated relationships between PA as assessed by exposure to vertical impacts and body mass index (BMI) in three population-based cohorts of older adults included in the Vertical Impacts on Bone in the Elderly (VIBE) study: the Cohort for Skeletal Health in Bristol and Avon (COSHIBA), Hertfordshire Cohort Study (HCS), and the Medical Research Council (MRC) National Survey of Health and Development (NSHD). The overarching aim of VIBE was to investigate the health benefits associated with higher impact PA across different population-based cohorts to optimize power and generalizability of findings. The aim of the present study was to examine whether more intense PA is associated with lower BMI, when intensity is based on vertical impact as opposed to cpm thresholds. We also examined to what extent differences in fat or lean mass contributed to observed relationships with BMI, based on contemporaneous dual X-ray absorptiometry (DXA)-derived measures of body composition collected in COSHIBA.

## Methods

### Study Populations

COSHIBA is a representative population-based cohort of 3200 women recruited through 15 general practices in the Bristol and Avon area during 2007–2009, originally set up to investigate determinants of skeletal health in postmenopausal women (11). Only 1286 COSHIBA participants who consented to be contacted about future research studies in 2014 and remained resident in the Bristol and Avon area were eligible to participate in the VIBE study. NSHD is a nationally representative sample of 5362 singleton births from 1 week in March 1946 (12,13). Most participants (79%) included in the home visit phase of the NSHD 24th data collection (2015–2016) (14) were invited to participate in the VIBE study. HCS comprises 3225 singleton births in Hertfordshire between 1931 and 1939 and still lived in the area during 1998–2003 (15). Only 443 HCS participants who were previously included in the UK arm of the European Project on Osteoarthritis (EPOSA) (16) were invited to participate in VIBE. In total, 3640 participants from the three cohorts were invited to participate in VIBE.

Separate regional ethical approval was obtained for data collection in NSHD (14/LO/1073 and 14/SS/1009), HCS (10/HO311/59), and COSHIBA (14/SW/0138) and written informed consent was obtained from all participants.

### PA Vertical Impacts From Accelerometers

Participants who were invited and agreed to accelerometry monitoring, subject to availability of monitors, were provided with a GCDC X15-1c triaxial accelerometer (Gulf Coast Data Concepts, Waveland, Mississippi), custom-designed size specific elasticated belt, a time log, and a stamped addressed package along with written and, if seen in clinic or during a nurse home visit, verbal instructions. Accelerometers were configured with standardized settings prior to participant use with a sampling frequency of 50 Hz, a deadband setting of 0.1*g* (the threshold which must be exceeded before a recording is made) and a timeout setting of 10 seconds (a single sample every 10 seconds is forced even if the recording is <0.1*g*). Participants were instructed to wear the accelerometer securely positioned in the belt over their right hip pointing toward the centre of their body for 7 continuous days, removing only for sleeping, washing, and swimming. A time log was provided for participants to record when the monitor was put on in the morning and taken off at night for each monitoring day and to state if there was any reason why that day had not been reflective of their normal activity.

Raw triaxial accelerometry data were uploaded to a secure shared drive and read into Stata 13 (StataCorp, College Station, Texas) for standardized cleaning and processing by the coordinating centre, described in detail elsewhere (7). In short, vertical (Y) axis accelerations data were cleaned to remove movement artifacts and nonwear time. Nonwear time was determined through prolonged sequences of zero movement readings lasting longer than 20 minutes and cross-checked against time sheets (7). Activity data were normalized for wear time based on 7 valid days (≥10 hours recording time) of 14 hours. Y-axis peaks were calculated based on accelerations higher than the preceding and subsequent reading and recorded within 14 pre-specified g bands. These were condensed to group all participants into three distinct impact bands to reflect low (0.5 < *g* <1.0), medium (1.0 < *g* < 1.5*g*), and higher (≥1.5*g*) impact. The ≥1.5*g* cut-point for higher impacts was selected as very few impacts were observed within higher *g* bands (7,9,17). Periods of inactivity were removed by excluding movements producing ≤0.5*g* (7). All *g* values represent *g* over and above 1*g* from earth’s gravitational force.

In addition to accelerometer-based measures of vertical impacts, we used concurrent participant reports of time spent during the past 7 days in different PA types (reported as “less than an hour,” “1–2 hours,” “2–4 hours,” or “more than 4 hours”) to derive conventional estimates of light (LPA) and moderate-to-vigorous intensity PA (MVPA) by assigning each PA a MET value (18). LPA included walking, bowls, Tai Chi, yoga, Pilates, housework, and light gardening, and MVPA comprised cycling, aerobics, aqua aerobics, badminton, dancing, football, hockey, water sports, tennis, squash, swimming, snow skiing, jogging, running, hiking, gym, golf, and heavy gardening.

### BMI

BMI (kg/m^2^) was calculated from heights and weights collected from each cohort. In COSHIBA, standing height was measured to the nearest millimetre using a Harpenden stadiometer (Holtain Ltd., Crymych, UK) and weight was measured to the nearest 50*g* using Tanita weighing scales (Tanita UK Ltd., Uxbridge, UK). Heights and weights were collected by questionnaire from HCS participants and were cross-checked against measured heights and weights from earlier ages for consistency. Finally, as part of the same home visit when the accelerometers were distributed to NSHD participants, nurses measured standing height to the nearest millimetre using a Leicester stadiometer (Marsden Group, UK) and weight was measured to the nearest 100*g* using Tanita weighing scales (Tanita UK Ltd., Uxbridge, UK).

### Fat and Lean Mass

All COSHIBA participants who attended the outcome assessment clinic were offered a DXA scan on a GE Healthcare Lunar Prodigy. Consenting participants who were able to transfer onto the DXA scan-bed unaided underwent a total body scan, generating fat and lean mass, and android:gynoid fat mass ratio. Repeat total body and hip DXA scans were performed in 20 COSHIBA participants within 1 month of the original scan. Within subject coefficients of variation were 1.7% for fat mass and 1.5% for lean mass indicating little variability.

### Covariates

Self-rated health, socioeconomic and functional status, and mental well-being, collected via questionnaire at the time of accelerometer monitoring, were identified as potential confounders and included as adjustments. Self-rated health was reported from very good to very poor in HCS and COSHIBA and from excellent to poor in NSHD. All participants reported their highest educational level by the age of 26, and the main occupation during working life from each participant and their spouse (if married) was assigned a 1990 Standard Occupational Classification (SOC90) code to obtain a proxy measure of social class. The highest SOC90 code between the participant and spouse was assigned where both occupations were provided. Functional status was indicated by walking speed (reported as unable to walk, very slow, stroll at an easy pace, normal speed, fairly brisk, or fast), whether ability to walk was restricted due to pain, and whether participants had a noticeable limp (difficulty walking). Mental well-being was based on the total score derived from responses to the Warwick-Edinburgh Mental Well-being Scale (19).

### Statistical Analysis

Linear regression models fit separately for each cohort, and each PA exposure were used to examine associations between accelerometer-based measures of low, medium, and higher impact PA and BMI. We used interaction terms to test sex differences (HCS, NSHD), and subsequently men and women were combined with adjustment made for sex after no evidence of interaction was found. We then combined studies and used interaction terms to test for cohort differences, and subsequently combined estimates from each cohort with random-effects meta-analysis after interactions were found between impacts and cohort. We used the *I*^2^ statistic to quantify between study heterogeneity in the summary random-effects estimates.

Initial models were adjusted for age (COSHIBA, HCS) and/or sex (HCS, NSHD) and, to examine the role of confounding, subsequent models were further adjusted for educational level, occupational class, self-rated health, walking speed, symptoms of pain during walking, difficulty walking (limping), and mental well-being. To explore whether associations were driven by a specific PA impact level, final models were fitted with additional mutual adjustment for all impact bands, after performing analyses to detect the degree, and investigate any potential consequence, of multicollinearity.

We then examined how impact magnitude relates to body composition in COSHIBA, firstly by plotting mean fat and lean mass across quartiles of low, medium, and higher impacts, and testing trends using an extension of the Wilcoxon rank-sum test (Cuzick’s test for trend). Subsequently, separate linear regression models were used to examine associations between the number of low, medium, and high impacts and fat and lean mass in COSHIBA. Initial models were adjusted for age and height, and, to examine the role of confounding, subsequent models were further adjusted for educational level, occupational class, self-rated health status, walking speed, symptoms of pain during walking, difficulty walking, and mental well-being. To investigate if findings for a certain PA impact magnitude were independent of other impact levels, final models included added mutual adjustments for all three PA impact bands.

To minimize the potential for bias due to missing data, we used multiple imputation by chained equations (20) to impute missing data for all covariates in participants with complete data on PA and BMI (*N* = 204 participants in total; education: *n* = 50, occupation: *n* = 67, self-rated health: *n* = 42, walking speed: *n* = 8, pain during walking: *n* = 30, difficulty walking: *n* = 36, and mental well-being: *n* = 52). Imputation models were run using 20 multiply imputed datasets which were combined using Rubin’s combination rules. Similar imputation models were used for body composition analyses in COSHIBA participants with complete data on PA and fat/lean mass. Imputed results were comparable to complete case analysis and the former are presented.

Accelerometer data were log-transformed due to their skewed distributions, and all outcomes (BMI/fat and lean mass) were standardized to a mean of 0 and standard deviation (*SD*) of 1. For each PA exposure, model estimates (ie *SD* change in outcomes per one log unit increase in impacts) were exponentiated to the power of 2 and interpreted as *SD* changes in outcomes per doubling in the number of impacts. For all outcomes, we examined nonlinearity using quadratic terms for impacts and fractional polynomial regression, but no evidence of nonlinear associations was found. Finally, we used a similar modeling strategy to examine associations between self-reported time spent in LPA and MVPA and BMI in all cohorts and fat and lean mass in COSHIBA.

## Results

### Participant Characteristics

Recruitment into the VIBE study for the three cohorts is shown in Supplementary Figure 1. A total of 1182 participants were included in analysis; mean age was 72.7 years (*SD* = 4.5) and 68% were female (Table 1). As previously reported (17), more low, medium, and high vertical impacts were observed in NSHD than in COSHIBA or HCS, reflecting their younger age and the higher proportion of males. As shown in Table 1, the majority of impacts were recorded within the low impact band than within the medium or high bands, representing 95.4%, 4.1%, and 0.5% of total impacts, respectively, in all cohorts combined. The numbers of impacts within different bands were moderately to highly correlated (eg spearman rank correlation coefficients in all cohorts combined were 0.9 for low vs medium impacts; 0.8 for medium vs higher impacts, and 0.7 for low vs higher impacts). Further analyses revealed no evidence that multicollinearity affected estimates; for example, variance inflation factors were 3.59, 7.17, and 3.53 for low, medium, and high impacts, respectively. Characteristics of participants according to the selected confounders are provided in Supplementary File 1.

**Table 1.** Key Characteristics of Participants From COSHIBA, HCS, and MRC NSHD, 2015

	COSHIBA (*N* = 430)	HCS (*N* = 104)	MRC NSHD (*N* = 648)	Combined (*N* = 1182)
Age (years) (mean [*SD*])	76.8 (3.0)	78.6 (2.6)	69 (0)	72.7 (4.5)
% female	100	39.4	48.8	66.6
BMI (kg/m^2^) (mean [*SD*])	27.1 (4.7)	25.6 (3.4)	27.3 (4.2)	27.1 (4.3)
Accelerometer wear time (days) (mean [*SD*])	5.2 (1.8)	4.2 (2.2)	4.9 (2.1)	5.1 (2.0)
Vertical acceleration peaks (median [IQR])				
Low impact (0.5 ≤ *g* < 1.0)	8687 (16624)	6940 (10903)	14765 (17453)	11573 (15826)
Medium impact (1.0 ≤ *g* <1.5)	340 (630)	227 (626)	811 (1866)	494 (1349)
Higher impact (≥ 1.5*g*)	42 (88)	40 (107)	94 (238)	63 (147)

*Note:* COSHIBA: Cohort for Skeletal Health in Bristol and Avon. HCS: Hertfordshire Cohort Study. MRC NSHD: Medical Research Council National Survey of Health and Development (the 1946 British birth cohort). BMI: body mass index. *SD*: standard deviation. IQR: interquartile range. Wear time based on ≥10 hours recording for any given day. Number of impacts within each band were normalized for wear time.

### Objectively Measured PA Impact Magnitude Versus BMI

**Figure 1.**
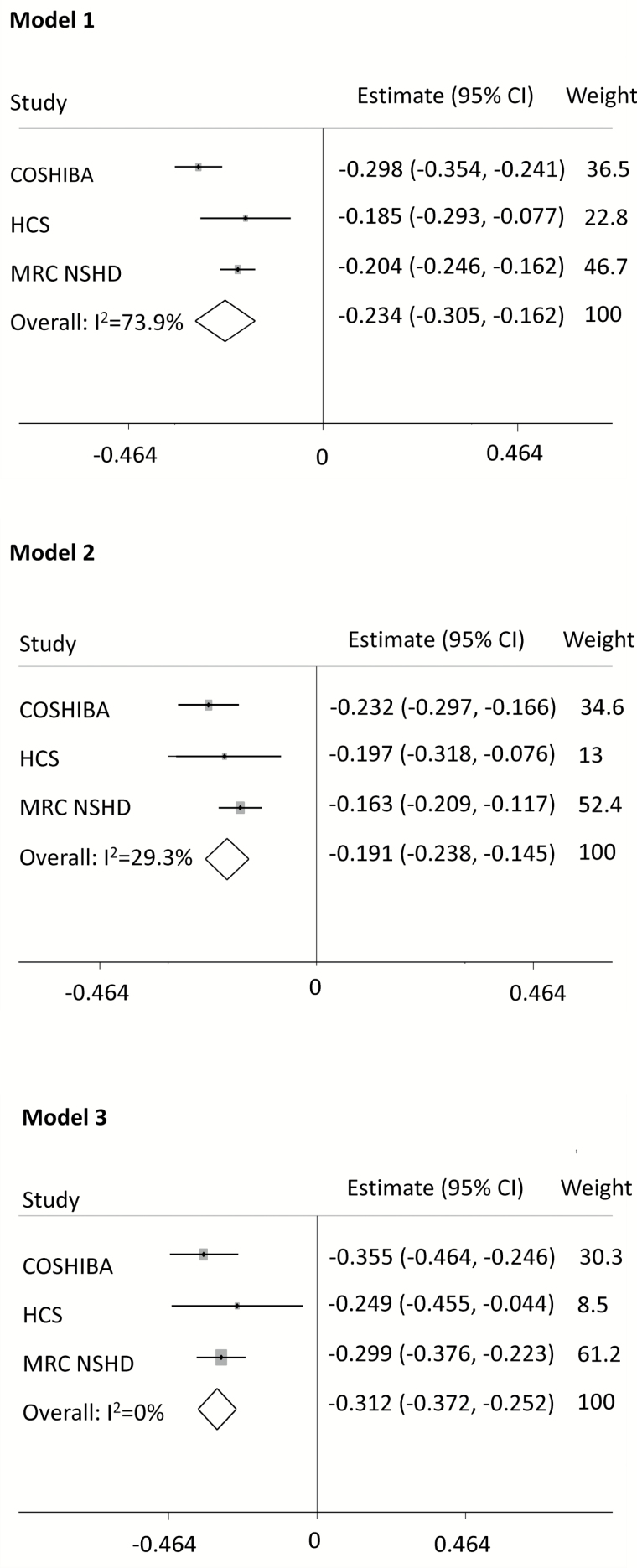
Standard deviation change in BMI per doubling in number of low magnitude vertical impacts (0.5 ≤ *g* < 1.0): random effects meta-analysis of results from COSHIBA, HCS, and MRC NSHD, 2015. COSHIBA: Cohort for Skeletal Health in Bristol and Avon. HCS: Hertfordshire Cohort Study. MRC NSHD: Medical Research Council National Survey of Health and Development. BMI: body mass index. Model 1: adjusted for age (COSHIBA and HCS) and sex (HCS and MRC NSHD). Model 2: additional adjustment for educational level, occupational class, self-rated health, walking speed, symptoms of pain during walking, difficulty walking, and mental well-being. Model 3: as for model 2 plus adjustment for medium and high impacts.

**Figure 2.**
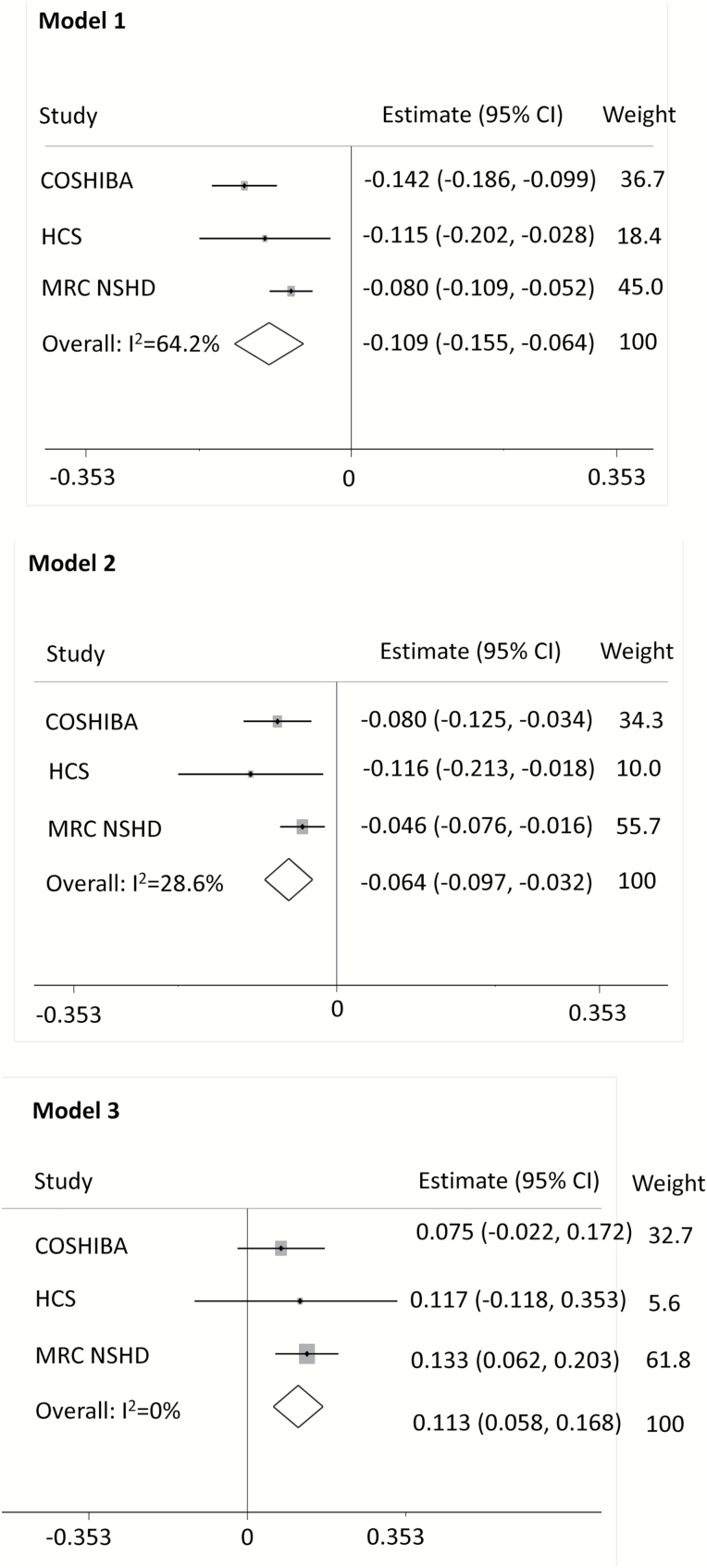
Standard deviation change in BMI per doubling in number of medium magnitude vertical impacts (1.0 ≤ *g* <1.5): random effects meta-analysis of results from COSHIBA, HCS, and MRC NSHD, 2015. COSHIBA: Cohort for Skeletal Health in Bristol and Avon. HCS: Hertfordshire Cohort Study. MRC NSHD: Medical Research Council National Survey of Health and Development. BMI: body mass index. Model 1: adjusted for age (COSHIBA and HCS) and sex (HCS and MRC NSHD). Model 2: additional adjustment for educational level, occupational class, self-rated health, walking speed, symptoms of pain during walking, difficulty walking, and mental well-being. Model 3: as for model 2 plus adjustment for low and high impacts.

**Figure 3.**
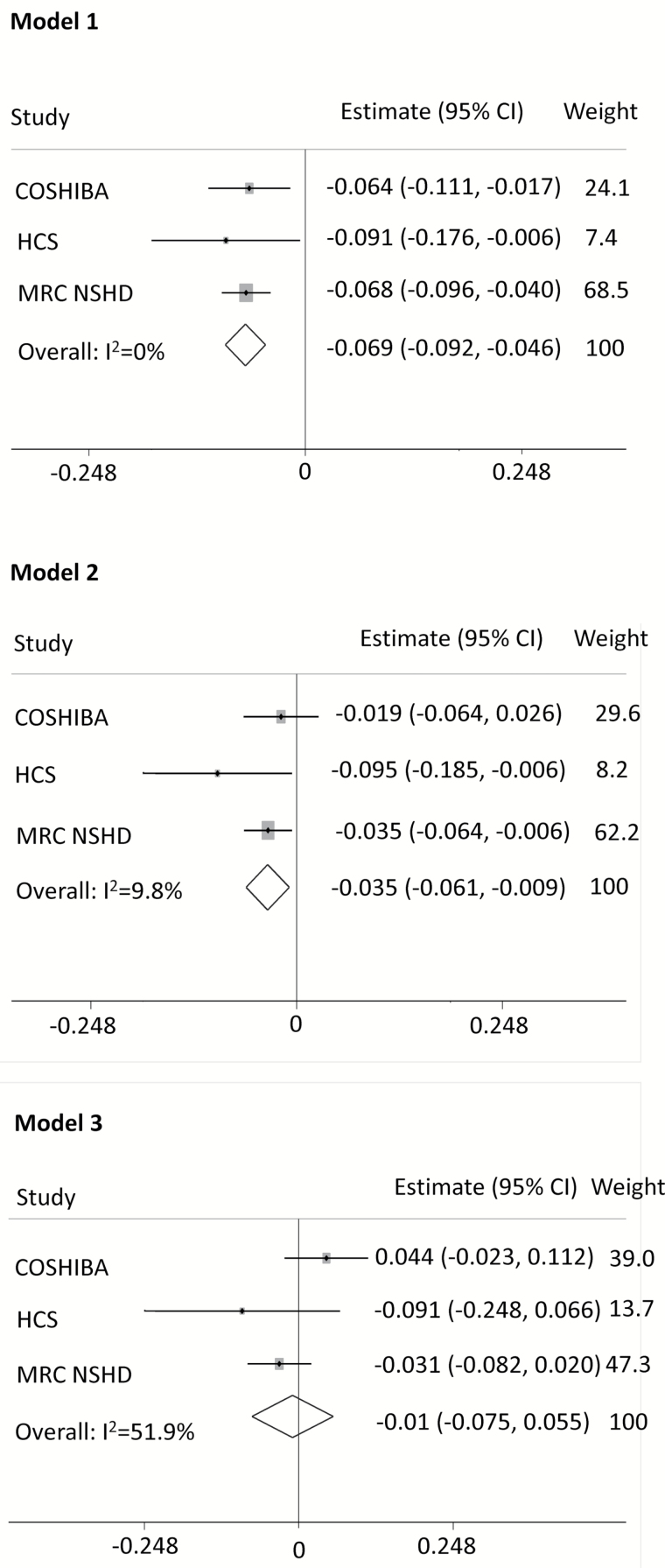
Standard deviation change in BMI per doubling in number of high magnitude vertical impacts (≥ 1.5*g*): random effects meta-analysis of estimates from COSHIBA, HCS, and MRC NSHD, 2015. COSHIBA: Cohort for Skeletal Health in Bristol and Avon. HCS: Hertfordshire Cohort Study. MRC NSHD: Medical Research Council National Survey of Health and Development. BMI: body mass index. Model 1: adjusted for age (COSHIBA and HCS) and sex (HCS and MRC NSHD). Model 2: additional adjustment for educational level, occupational class, self-rated health, walking speed, symptoms of pain during walking, difficulty walking, and mental well-being. Model 3: as for model 2 plus adjustment for low and medium impacts.

In initial (age and/or sex-adjusted) models, greater numbers of low, medium, and higher impacts were each associated with lower BMI in all three cohorts, with differences becoming progressively weaker on an increasing impact level (Figures 1–3). Results were unaffected by further adjustment for confounders (Figures 1–3). Following additional mutual adjustment for all impacts levels, only low impacts were inversely related to BMI, whereas a positive association with BMI was found for medium impacts (Figures 1–3). Associations between low impacts and BMI appeared stronger in COSHIBA, but only in initial models (*I*^2^=73.9%), and in all three cohorts, the main direction of association was the same (Figure 1).

### Objectively Measured PA Impact Magnitude Versus Fat and Lean Mass in COSHIBA

**Figure 4.**
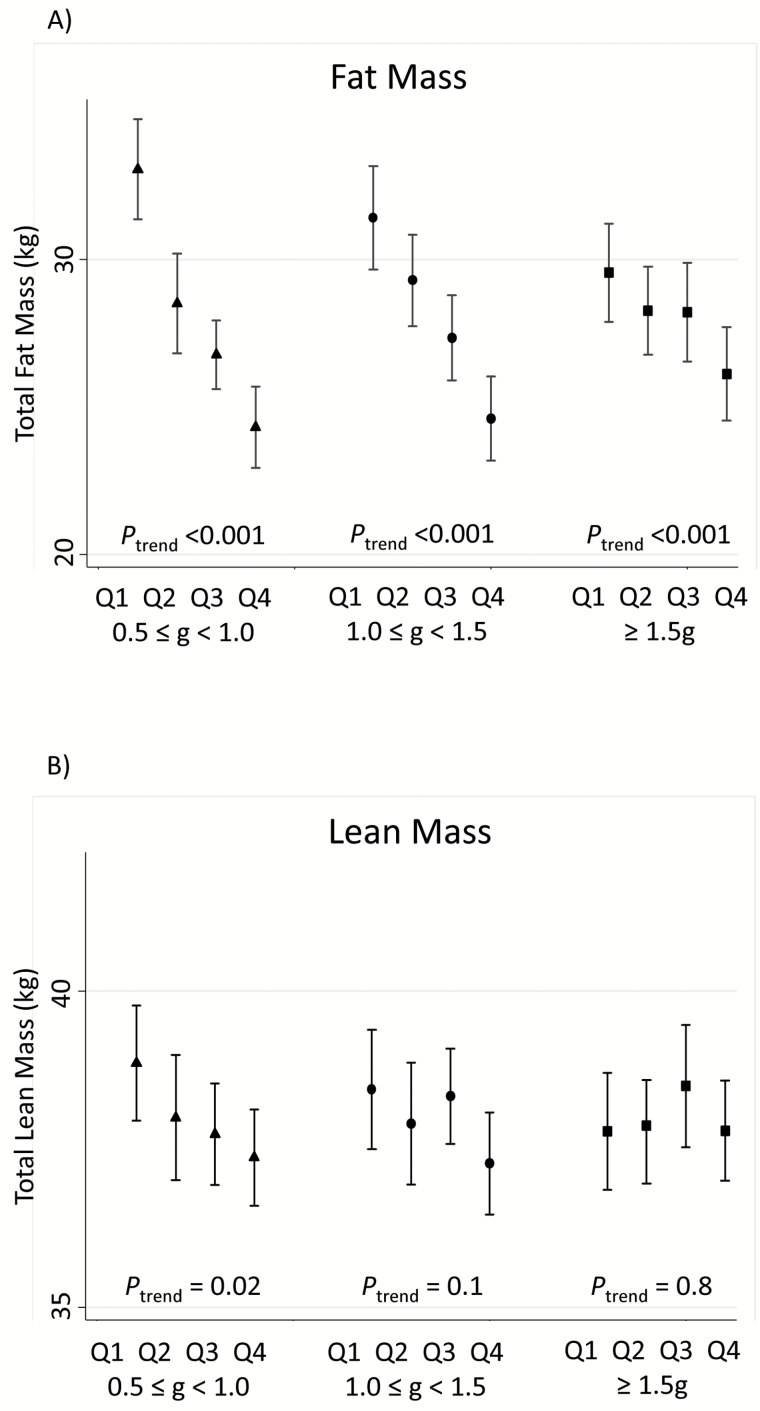
Mean total body (**A**) fat and (**B**) lean mass across quartiles of low, medium, and higher magnitude vertical impacts in COSHIBA. COSHIBA: Cohort for Skeletal Health in Bristol and Avon. *N* = 436 (*n* = 109 in each quartile). Range of number of vertical impacts for (a) low impacts: Q1 = 213 to 3969, Q2 = 3988 to 8534, Q3 = 8609 to 16487, Q4 = 16617 to 68779; (b) medium impacts: Q1 = 2 to 97, Q2 = 97 to 338, Q3 = 339 to 724, Q4 = 726 to 976; and (c) high impacts: Q1 = 0 to 17, Q2 = 17 to 41, Q3 = 41 to 105, Q4 = 105 to 4500. *p* for trend from Cuzick’s test for trend.

There were trends of lower total fat mass across higher quartiles of impacts for all impact bands; however, the difference progressively reduced on moving from low to medium to higher impacts (Figure 4). Similar associations were observed in models adjusted for age and height, with inverse associations between vertical impacts and fat mass becoming progressively weaker on an increasing impact level (Table 2). Results were unchanged after further adjustment for lean mass and selected confounders; however, following additional mutual adjustment for all PA impact levels, an inverse relationship between impacts and total fat mass was only seen for low impacts (Table 2). Similar findings were seen for android:gynoid fat mass ratio; for example, *SD* differences in android:gynoid fat mass ratio (95% confidence intervals) per doubling in the number of low, medium, and higher impacts were −0.16 ([−0.29, −0.03], *p* = .01), 0.02 ([−0.09, 0.13], *p* = .7), and −0.02 ([−0.10, 0.06], *p* = .7), respectively (adjusted for height, age, lean mass, educational level, occupational class, self-rated health, walking speed, symptoms of pain during walking, difficulty walking, mental well-being, and other PA impact levels).

**Table 2.** Standard Deviation Change in Total Body Lean and Fat Mass per Doubling in Number of Low (0.5 ≤ *g* < 1.0), Medium (1.0 ≤ *g* <1.5), and Higher (≥ 1.5*g*) Magnitude Vertical Impacts in COSHIBA Participants, 2015 (*N* = 425).

	Model 1 (95% CI)	*p*	Model 2 (95% CI)	*p*	Model 3 (95% CI)	*p*
Total body lean mass						
Low impact	−0.18 (−0.23, −0.13)	<.001	−0.02 (−0.08, 0.03)	.4	−0.09 (−0.18, 0.00)	.05
Medium impact	−0.07 (−0.11, −0.03)	<.001	0.01 (−0.02, 0.05)	.4	0.02 (−0.06, 0.09)	.7
Higher impact	−0.01 (−0.05, 0.03)	.7	0.04 (0.00, 0.07)	.03	0.05 (0.00, 0.11)	.06
Total body fat mass						
Low impact	−0.29 (−0.35, −0.24)	<.001	−0.16 (−0.21, −0.10)	<.001	−0.19 (−0.29, −0.10)	<.001
Medium impact	−0.14 (−0.19, −0.10)	<.001	−0.07 (−0.11, −0.03)	<.001	0.03 (−0.05, 0.12)	.4
Higher impact	−0.08 (−0.12, −0.03)	.002	−0.05 (−0.08, −0.01)	.02	−0.01 (−0.06, 0.05)	.8

*Note:* COSHIBA: Cohort for Skeletal Health in Bristol and Avon. Estimates show standard deviation change in lean and fat mass per doubling in number of vertical acceleration peaks within each impact band. Model 1: adjusted for height and age. Model 2: additional adjustment for fat/lean mass, educational level, occupational class, self-rated health, walking speed, symptoms of pain during walking, difficulty walking, and mental well-being. Model 3: as for model 2 plus adjustment for other impact bands.

Higher quartiles of low impacts were inversely associated with total lean mass; however, no associations were found for medium or higher impacts (Figure 4). In age- and height-adjusted analyses, low and medium impacts were inversely related to total lean mass; however, all levels of impact were unrelated to lean mass following additional adjustment for confounders (Table 2). Further investigation revealed that this attenuation was driven by adjustment for fat mass. Following additional mutual adjustment for impacts in other bands, there was an inverse association between low impacts and lean mass, and a weak positive association between higher impacts and lean mass (Table 2).

### Self-Reported LPA and MVPA Versus BMI and Fat and Lean Mass

LPA and MVPA were inversely related to BMI in all cohorts including after adjustments, and associations appeared stronger for LPA than MVPA (see Supplementary Table 2). In analyses based on COSHIBA, LPA and MVPA were inversely related to fat mass and android:gynoid fat mass ratio but differences appeared larger for MVPA than LPA (see Supplementary Table 3). LPA and MVPA were both inversely related to lean mass in initial models; however, after adjustment for confounders and mutual adjustment for reported PA intensity, MVPA was positively related to lean mass, whereas no association was observed for LPA (see Supplementary Table 3).

## Discussion

We examined associations between habitual PA as assessed by exposure to vertical impacts and BMI in three population-based cohorts of older adults. Low, medium, and higher impact PAs were initially associated with lower BMI; however, only low impact PA was inversely related to BMI after mutual adjustment for all impact bands. Low, but not medium or higher, impacts were also inversely associated with fat mass and android:gynoid fat mass ratio, based on DXA-derived measures available in COSHIBA. In contrast, higher, but not medium or low, impacts were positively associated with total body lean mass. On comparing these findings with those from application of MET thresholds to self-reported PA, equivalent positive relationships were observed for MVPA and lean mass, whereas MVPA was inversely related to fat mass and android:gynoid ratio. Taken together, these findings suggest that when evaluating relationships between more intense PA and body composition in older adults, equivalent positive associations were seen with lean mass, irrespective of whether PA was evaluated by measurement of vertical impacts or using MET-based approaches. In contrast, whereas MVPA was inversely associated with fat mass, inverse relationships between impacts and fat mass became attenuated at higher impact levels.

Accelerometer measures in the present study were designed to capture information about individual vertical impacts, to which the skeleton is thought to preferentially respond (7). This represents a distinct approach to the more commonly used Actigraph cpm method, in which impact frequency and magnitude are amalgamated. Nonetheless, the number of impacts is expected to show some relationship with conventional PA intensity measures based on energy consumption, which may have contributed to the inverse associations we observed between low impacts and BMI and fat mass. Consistent with this, low impacts were positively related to both LPA and MVPA (both 0.08 [0.07, 0.10]; *SD* increase in LPA/MVPA per *SD* increase in low impacts).

In contrast, neither medium nor higher impacts were related to these obesity outcomes after adjusting for low impacts, suggesting that vertical impacts per se have little direct relationship with obesity. A similar lack of association between high vertical impacts and fat mass was reported in our previous study of adolescents (21). One explanation for the lack of inverse associations between high impacts and BMI and fat mass could be that short bouts of activities involving high impacts like jumping up and down for a few seconds are unlikely to produce noteworthy effects on energy expenditure, unless sustained for longer periods. Consistent with this, associations may have been observed for low impact PA since these make up the majority of time spent in PA in later life. Alternatively, the null association between medium and higher impacts and obesity outcomes may reflect a lack of power, given that considerably fewer counts were recorded as compared with low impacts. That said, our study did have sufficient power to detect positive associations between higher impacts and lean mass using equivalent adjustments.

The lack of association between higher vertical impacts and BMI and fat mass contrasts with the case when PA intensity is categorized according to energy expenditure. As expected, MVPA as assessed by self-report showed a stronger inverse association with fat mass and android:gynoid fat mass ratio compared to LPA in COSHIBA. Equivalent results were found in NSHD participants at the age of 60–64 years using objective PA measurement with the chest-worn actiheart monitor (1). However, if anything, self-reported LPA was related to BMI more strongly than MVPA. This apparent discrepancy may reflect the difficulty in using questionnaires to evaluate PA intensity, especially since walking encompasses both LPA and MVPA (18). Consistent with this possibility, in sensitivity analyses in which walking activity was assigned to MVPA rather than LPA, MVPA showed a strong inverse association with BMI in the three cohorts combined (−0.14 [−0.20, −0.09], *p* < .001), whereas LPA was only weakly associated (−0.04 [−0.009, 0.01], *p* = .1; *SD* change in BMI per *SD* increase in LPA/MVPA).

Aerobics and dancing are the most common activities to generate higher vertical impacts in the three cohorts contributing to this study (17). Hence, the present findings would seem to suggest that participation in these activities is not protective against obesity, implying that whereas relatively short bouts of higher impact PA may be beneficial for bone and muscle health in older people (9), sustained amounts of low impact PA may be more important in combatting obesity. However, an important caveat is that exposure to higher impacts as assessed in this study is also likely to reflect intrinsic factors such as muscle strength and tendon elasticity, as opposed to participation in specific activities. Consistent with this suggestion, we previously reported that the number of higher vertical impacts shows relatively weak relationships with participation in specific activities producing high impacts (17); furthermore, in our preliminary analyses, gait speed appears to be the strongest predictor of high impacts (our unpublished observations).

In contrast to our findings with fat mass, more intense PA was associated with greater lean mass irrespective of how PA intensity was assessed; in our fully adjusted model, high impacts and MVPA were both positively associated with lean mass, whereas equivalent relationships were not seen for low/medium impacts or LPA. These findings are likely to reflect a positive relationship between participation in more intense PA and underlying muscle function, given previous reports that MVPA in older individuals as assessed by accelerometry is positively related to muscle size and strength (22,23).

### Strengths and Limitations

A strength of this study is our use of a novel accelerometer-based method to extract parameters from raw data to provide new insights into the health benefits of PA (24–26). Other strengths include the use of three population cohorts encompassing a broad age range of older individuals, employing identical protocols for objectively evaluating exposure to vertical impacts, minimizing bias due to missing data by including participants with incomplete information on covariates, and utilizing DXA body composition outcomes and cross-evaluation of accelerometer results with self-reported PA (2,27).

In terms of limitations, use of a cross sectional study design makes it difficult to exclude reverse causality for which some evidence exists in the case of PA-obesity relationships (28,29). Furthermore, although accelerometry is useful in providing an objective measure of PA, this method has several inherent limitations. For example, PA levels are likely to be affected by seasonal influences, although data collection was avoided during prolonged periods of ice and snow which would have significantly restricted outdoors activity. However, on further exploration, there was little evidence that accelerometry counts within the three bands differed by season (17). Arguably, it would have been preferable to derive estimates of LPA and MVPA from accelerometer recordings; however, this was not possible since the GCDC devices used in this study turn off when no movement is detected, to save battery life. Selection bias is possible as VIBE participants tended to have lower BMI and higher educational level compared with others who did not participate in VIBE (17), consistent with selection effects; however, this would have underestimated the associations observed. Finally, residual confounding due to unmeasured confounders or measurement error in the measured confounders might influence our findings.

## Conclusions

We examined associations between habitual PA as assessed by exposure to vertical impacts and BMI in three cohorts of older people. We found that low vertical impacts were inversely related to BMI and fat mass; however, equivalent independent relationships were not seen for medium or higher impacts. This contrasts with results when PA estimates were based on energy consumption, since LPA and MVPA were both found to be inversely related to BMI and fat mass. Thus, whereas high impact PA may be beneficial for bone health at older age, strategies to increase exposure of older people to lower impact activities such as walking and swimming may provide the greatest benefit for reducing obesity risk.

## Supplementary Material

Supplementary data is available at *The Journals of Gerontology, Series A: Biological Sciences and Medical Sciences* online.

## Funding

This work was supported by the UK Medical Research Council [grant number: MR/K024973/1].

## Author Contributions

Study design: JT, KH, EMC, CC, DK. Study conduct and data collection: KH, KCD, AH, CM, MHE, ED, TG, AW, RC. Data analysis: AE. Data interpretation: JT, AE, RC. JT produced the first manuscript draft and all authors read and approved its final version.

## Conflict of Interest

None reported.

## Supplementary Material

Supplementary_table1

Supplementary_figure1

Supplementary_table3

Supplementary_table2
