# Supplementary material for: Physical Activity Producing Low, but Not Medium or Higher, Vertical Impacts Is Inversely Related to BMI in Older Adults: Findings From a Multicohort Study
Source: J Gerontol A Biol Sci Med Sci. 2017 Sep 19;73(5):643–51. doi: 10.1093/gerona/glx176 (PMC5846734; doi:10.1093/gerona/glx176)
Supplement: Supplementary_table1 [file glx176_suppl_supplementary_table1.pdf]

Supplementary table 1. Characteristics of participants from COSHIBA, HCS and MRC NSHD, 2015.

	COSHIBA (n=430)	HCS (n=104)	MRC NSHD (n=648)	Combined (n=1182)
Educational level by age 26				
None	175 (42.6)	60 (60.0)	169 (27.2)	404 (35.7)
Up to and including O level	138 (33.6)	27 (27.0)	169 (27.2)	334 (29.5)
A level & above	98 (23.8)	13 (13.0)	283 (45.6)	394 (34.8)
Highest SOC90				
1-2	200 (49.8)	35 (35.4)	333 (54.2)	568 (50.9)
3-4	128 (31.8)	24 (24.2)	169 (27.5)	321 (28.8)
5-6	55 (13.7)	22 (21.2)	83 (13.5)	160 (14.3)
7-8	19 (4.7)	18 (18.2)	29 (4.7)	66 (5.9)
Self-rated health				
Very good or excellent	106 (24.9)	16 (15.8)	354 (57.7)	476 (41.8)
Good	237 (55.8)	60 (59.4)	191 (31.1)	488 (42.8)
Fair or poor/very poor	82 (19.3)	25 (24.8)	69 (11.2)	176 (15.4)
Walking speed				
Unable to walk or very slow	34 (7.9)	8 (7.7)	15 (2.3)	57 (4.9)
Stroll at an easy pace	100 (23.4)	35 (33.7)	89 (13.9)	224 (19.1)
Normal speed	191 (44.6)	46 (44.2)	337 (52.5)	574 (48.9)

Fairly brisk or fast	103 (24.1)	15 (14.4)	201 (31.3)	319 (27.2)
Ability to walk restricted due to pain				
No	265 (64.8)	67 (64.4)	516 (80.8)	848 (73.6)
Yes	144 (35.2)	37 (35.6)	123 (19.3)	304 (26.4)
Noticeable limp				
No	369 (87.9)	85 (83.3)	580 (93.0)	1034 (90.2)
Yes	51 (12.1)	17 (16.7)	44 (7.0)	112 (9.8)
Warwick-Edinburgh Mental Wellbeing Scale[a]	0.0 (1.0)	-0.1 (1.0)	0.0 (1.0)	0.0 (1.0)

COSHIBA: Cohort for Skeletal Health in Bristol and Avon. HCS: Hertfordshire Cohort Study. MRC NSHD: Medical Research Council National Survey of Health and Development (the 1946 British birth cohort). BMI: body mass index. SD: standard deviation. IQR: interquartile range. SOC90: 1990 Standard Occupational Classification. Self-rated health was reported from very good to very poor in HCS and COSHIBA and from excellent to poor in NSHD. [a]Standardised to mean of 0 and standard deviation of 1. Sample size varies depending on those with data on each characteristic (for analyses, multiple imputation is used to impute missing data on each characteristic).
