# Supplementary material for: Physical Activity Producing Low, but Not Medium or Higher, Vertical Impacts Is Inversely Related to BMI in Older Adults: Findings From a Multicohort Study
Source: J Gerontol A Biol Sci Med Sci. 2017 Sep 19;73(5):643–51. doi: 10.1093/gerona/glx176 (PMC5846734; doi:10.1093/gerona/glx176)

Supplementary figure 1. Recruitment of VIBE study participants from A) COSHIBA, B) HCS and C) MRC NSHD. VIBE: Vertical Impacts on Bone in the Elderly study. COSHIBA: Cohort for Skeletal Health in Bristol and Avon. HCS: Hertfordshire Cohort Study. MRC NSHD: Medical Research Council National Survey of Health and Development.

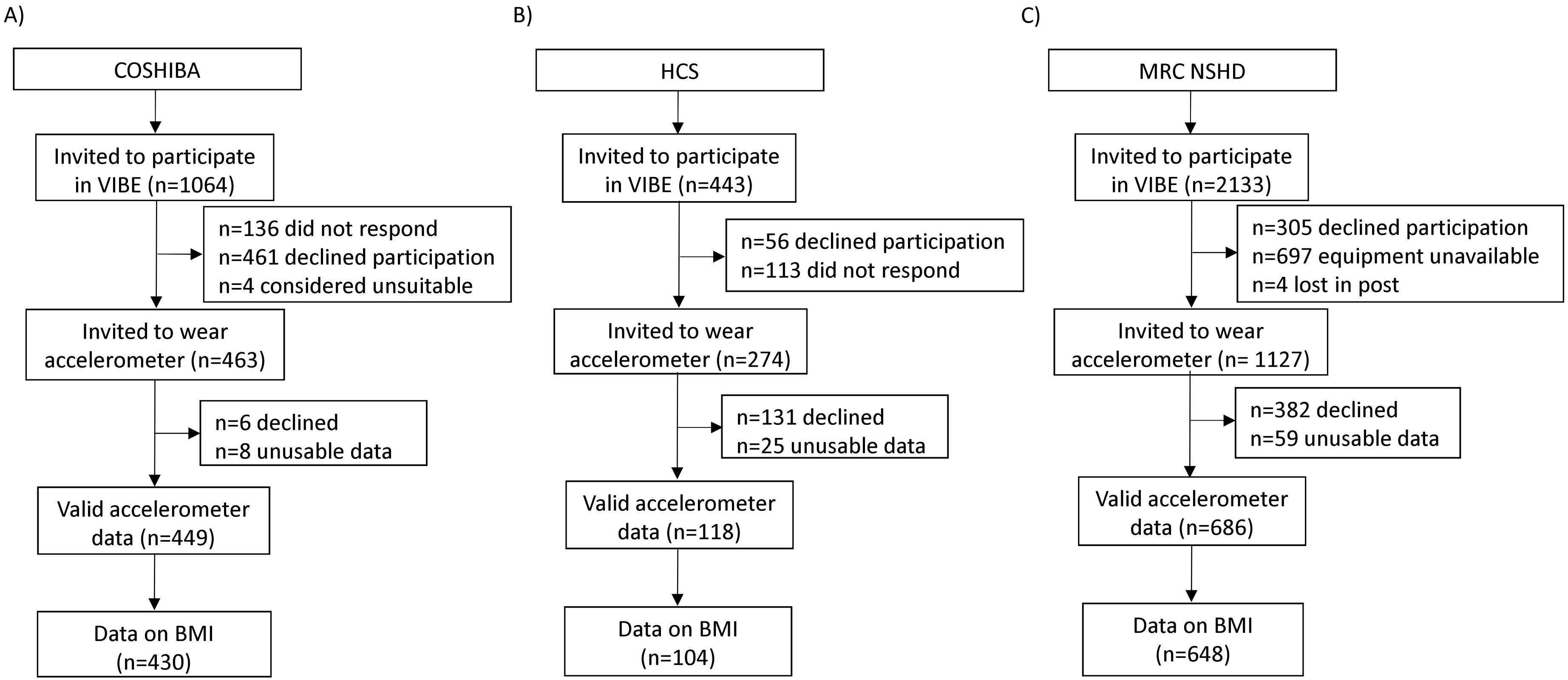

Supplement: Supplementary_figure1 [file glx176_suppl_supplementary_figure1.pdf]
