# Supplementary material for: Physical Activity Producing Low, but Not Medium or Higher, Vertical Impacts Is Inversely Related to BMI in Older Adults: Findings From a Multicohort Study
Source: J Gerontol A Biol Sci Med Sci. 2017 Sep 19;73(5):643–51. doi: 10.1093/gerona/glx176 (PMC5846734; doi:10.1093/gerona/glx176)
Supplement: Supplementary_table3 [file glx176_suppl_supplementary_table3.pdf]

Supplementary table 3. Self-reported LPA and MVPA and lean and fat mass in COSHIBA, 2015 (n=458).

	Model 1 (95% CI)	*P*	Model 2 (95% CI)	*P*	Model 3 (95% CI)	*P*
Total body lean mass						
LPA	-0.11 (-0.20, -0.03)	0.007	0.05 (-0.02, 0.12)	0.1	0.03 (-0.04, 0.10)	0.4
MVPA	-0.09 (-0.19, 0.01)	0.07	0.12 (0.04, 0.20)	0.005	0.11 (0.02, 0.19)	0.01
Total body fat mass						
LPA	-0.28 (-0.37, -0.19)	<0.001	-0.17 (-0.24, -0.09)	<0.001	-0.13 (-0.20, -0.05)	0.001
MVPA	-0.33 (-0.43, -0.22)	<0.001	-0.22 (-0.30, -0.13)	<0.001	-0.18 (-0.27, -0.09)	<0.001
Android:gynoid fat mass ratio						
LPA	-0.16 (-0.25, -0.07)	0.001	-0.13 (-0.22, -0.03)	0.009	-0.08 (-0.18, 0.02)	0.1
MVPA	-0.27 (-0.39, -0.16)	<0.001	-0.23 (-0.34, -0.12)	<0.001	-0.20 (-0.32, -0.09)	0.001

COSHIBA: Cohort for Skeletal Health in Bristol and Avon. LPA: light intensity physical activity. MVPA: moderate-to-vigorous intensity physical activity. Estimates show standard deviation increase in outcomes per standard deviation increase in LPA and MVPA hours in past 7 days. Model 1: adjusted for height and age. Model 2: additional adjustment for fat/lean mass, educational level, occupational class, self-rated health, walking speed, symptoms of pain during walking, difficulty walking and mental wellbeing. Model 3: as for model 2 plus adjustment for LPA/MVPA.
