# Supplementary material for: Physical Activity Producing Low, but Not Medium or Higher, Vertical Impacts Is Inversely Related to BMI in Older Adults: Findings From a Multicohort Study
Source: J Gerontol A Biol Sci Med Sci. 2017 Sep 19;73(5):643–51. doi: 10.1093/gerona/glx176 (PMC5846734; doi:10.1093/gerona/glx176)
Supplement: Supplementary_table2 [file glx176_suppl_supplementary_table2.pdf]

Supplementary table 2. Self-reported LPA and MVPA and BMI in COSHIBA, HCS and MRC NSHD, 2015.

	Model 1 (95% CI)	*P*	Model 2 (95% CI)	*P*	Model 3 (95% CI)	*P*
COSHIBA (n=463)						
LPA	-0.27 (-0.35, -0.18)	<0.001	-0.16 (-0.25, -0.08)	<0.001	-0.14 (-0.24, -0.05)	0.002
MVPA	-0.28 (-0.39, -0.18)	<0.001	-0.13 (-0.23, -0.02)	0.02	-0.08 (-0.19, -0.03)	0.2
HCS (n=115)						
LPA	-0.13 (-0.33, 0.07)	0.2	-0.09 (-0.31, 0.12)	0.4	-0.07 (-0.29, 0.15)	0.5
MVPA	-0.16 (-0.33, 0.02)	0.08	-0.11 (-0.31, 0.09)	0.3	-0.09 (-0.30, 0.11)	0.4
MRC NSHD (n=684)						
LPA	-0.16 (-0.22, -0.10)	<0.001	-0.10 (-0.16, -0.03)	0.003	-0.08 (-0.15, -0.02)	0.01
MVPA	-0.12 (-0.18, -0.06)	<0.001	-0.07 (-0.13, -0.01)	0.02	-0.05 (-0.11, 0.01)	0.1

Combined (n=1262)						
LPA	-0.20 (-0.25, -0.15)	<0.001	-0.12 (-0.17, -0.07)	<0.001	-0.10 (-0.15, -0.05)	<0.001
MVPA	-0.17 (-0.22, -0.11)	<0.001	-0.09 (-0.14, -0.04)	0.001	-0.06 (-0.11, -0.01)	0.02

COSHIBA: Cohort for Skeletal Health in Bristol and Avon. HCS: Hertfordshire Cohort Study. MRC NSHD: Medical Research Council National Survey of Health and Development. BMI: body mass index. LPA: light intensity physical activity. MVPA: moderate-to-vigorous intensity physical activity. Estimates show standard deviation increase in BMI per standard deviation increase in LPA and MVPA hours in past 7 days. Model 1: adjusted for age (COSHIBA)/sex (NSHD)/age and sex (HCS)/age, sex and cohort (combined). Model 2: additional adjustment for educational level, occupational class, self-rated health, walking speed, symptoms of pain during walking, difficulty walking and mental wellbeing. Model 3: as for model 2 plus adjustment for LPA/MVPA.
